# Supplementary figures and images for: BCOR, BCORL1, and BCL6 Mutations in Pediatric Leukemias
Source: Cancers (Basel). 2025 Jul 23;17(15):2443. doi: 10.3390/cancers17152443 (PMC12345890; doi:10.3390/cancers17152443)

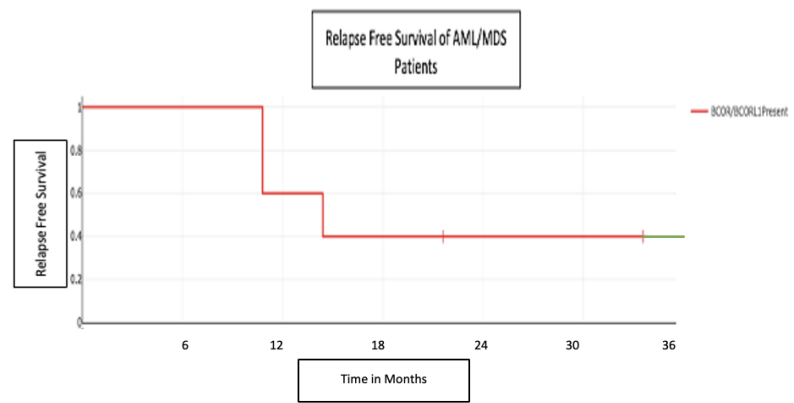

**Figure S1.** Kaplan–Meier survival curve for AML/MDS patients (n = 5).

Supplement: Supplementary file 1 [file cancers-17-02443-s001.zip › cancers-3718918-supplementary.pdf]
